# Comparison of diagnostic accuracy and sensitivity of Kinyoun and auramine-rhodamine stains in detecting mycobacteria from specimen smears

**DOI:** 10.1128/spectrum.00475-26

**Published:** 2026-05-19

**Authors:** Hafij Al Mahmud, Rosemary C. She

**Affiliations:** 1 Department of Pathology, City of Hope National Medical Center 20220, Duarte, California, USA; Institut de Pharmacologie et de Biologie Structurale, Toulouse, France

**Keywords:** fluorochrome, carbolfuschin, non-tuberculous mycobacteria, diagnostic accuracy

## Abstract

Smear microscopy using carbolfuschin- or auramine-based staining is widely employed for rapid mycobacterial detection from patient specimens. While auramine stains are preferred to detect *M. tuberculosis* complex (MTBC), there are little data on the relative performance of these stains for the detection of nontuberculous mycobacteria (NTM). This retrospective study evaluated the diagnostic accuracy of Kinyoun and auramine-rhodamine (AR) stains on clinical specimens from a tertiary cancer center (2017–2025), using culture-based identification as the gold standard. Of 16,294 specimens, 651 (4.0%) recovered mycobacteria, predominantly *Mycobacterium avium* complex (MAC) (56.2%) and MTBC (14.4%). Both stains demonstrated high specificity (>99.9%) but limited sensitivity (17.4% overall, 11.2%–11.8% among index cases) for mycobacteria, with higher sensitivity for MTBC (40.4%–43.6%) than NTM (12.9%–13.5%) (*P* < 0.0001). 1.7% of mycobacteria-positive specimens had discordant qualitative AR vs Kinyoun smear results: AR detected three MTBC and three MAC cases missed by Kinyoun stain, while Kinyoun stain detected five rapid-growing mycobacteria (RGM) cases not detected by AR, though differences were not statistically significant (*P* > 0.05). Semi-quantitatively (1-4+), of 107 AR+/Kinyoun+ smears, AR was one grade higher in 9 specimens (3 MTBC, 5 MAC, 1 RGM), while Kinyoun was one grade higher in 3 specimens (2 MAC, 1 RGM). A significant increase in frequency of NTM isolation in culture over the study period was observed *(P* = 0.02), but MTBC frequency remained stable. Given time and resource considerations, even where NTM predominates in prevalence, AR is appropriate as the primary mycobacterial stain, but carbolfuschin-based staining should be considered when RGM is suspected.

**IMPORTANCE:**

Accurate and rapid detection of mycobacteria is essential for timely patient management, especially in vulnerable immunocompromised patients. This study analyzes more than 16,000 clinical specimens over 8 years and provides a direct comparison of auramine rhodamine and Kinyoun stains for mycobacterial detection on smear microscopy. Both stains show high specificity and comparable overall performance, but Kinyoun exhibits a modest trend toward improved the detection of rapid growing mycobacteria. These findings offer guidance for clinical laboratories performing smear microscopy for both tubercular and non-tuberculous mycobacteria.

## INTRODUCTION

Mycobacterial infections including tuberculosis (TB) present diagnostic challenge which, in turn, pose significant obstacles toward the detection and eradication of mycobacterial disease (1). The World Health Organization (WHO) recommends low-complexity automated and manual nucleic acid amplification tests for the initial diagnosis of TB and smear microscopy and culture for monitoring response to treatment for pulmonary TB (2). The Centers for Disease Control and Prevention (CDC) includes sputum smear and culture in its recommendations for laboratory detection of tuberculosis (3). In the USA and other high-resource countries, nontuberculous mycobacterium (NTM) infections are more prevalent than *Mycobacterium tuberculosis* (MTB) infection and must also be considered when performing mycobacterial smear and culture. The CDC also recommends acid-fast bacilli (AFB) stain and bacterial culture in the diagnostic work-up for NTM (4). AFB-stained smears have been used as a cornerstone for rapidly detecting mycobacteria directly from patient specimens. Mycobacteria and some aerobic Actinomycetes retain certain dyes even after being treated with strong acid due to unique cell wall properties (5, 6). Two well recognized techniques used to visualize AFB under the microscope are carbolfuchsin and fluorochrome staining methods. Carbolfuchsin staining has evolved over the years and the well adapted and frequently used variants in clinical laboratories are Ziehl-Neelsen and Kinyoun stains (5, 7). Fluorochrome auramine-O (AO) or auramine-rhodamine (AR) are the frequently used fluorescent AFB staining techniques (5, 8). As a fluorescent stain, AR staining generally detects tubercle bacilli more rapidly and with greater sensitivity than carbolfuchsin-based methods though it is hindered by background fluorescence from non-specific artifact staining (5). While the exact basis of the acid-fast staining is unclear, studies using genetically modified MTB stains suggest that mycolic acids and other cell wall glycolipids are key elements to this property (9). Despite their widespread use, carbolfuchsin and fluorochrome staining methods can be affected by factors such as bacillary dormancy, mycolic acid synthesis inhibitors (e.g., isoniazid), staining time, microscopy settings, low bacterial load, and operator variability (9).

The CDC recommends using AR or AO staining for primary specimens and carbolfuchsin staining for confirming culture isolates as AFB (10). Compared to carbolfuchsin, fluorescence-based methods are generally 10% higher in diagnostic sensitivity in detecting pulmonary tuberculosis (11, 12). However, conflicting results have been reported for the relative performance of these staining techniques with NTM, and recommendations to consider carbolfuschin staining in smear microscopy for RGM are based on few published studies (12, 13). While the AR stain has also been reported to have low sensitivity for detecting RGM (13), AO was found to be more sensitive compared to ZN in detecting NTM from histologically prepared tissue (14), with little other published data comparing AO/AR to carbolfuscin staining in detecting NTM from specimen smears. Given the inconsistent findings and small number of reported studies in the literature, we sought to evaluate the sensitivity of AR staining relative to the Kinyoun method for detecting MTBC and NTM including RGM from direct specimens. We further sought to assess the performance of AFB smear microscopy relative to culture across the breadth of mycobacterial species that may be recovered from immunocompromised patients. City of Hope specializes in cancer care for a mostly adult patient population with services including hematopoietic stem cell transplant, chemotherapy, chimeric antigen receptor T-cell therapy, gene therapy, and clinical trials research. This specialized patient cohort offers a distinct opportunity to assess mycobacterial diagnostic performance in high-risk clinical settings At our tertiary care cancer center, both Kinyoun and AR staining have been routinely performed together for AFB smear microscopy to maximize the detection of AFB including RGM, providing a unique opportunity to directly compare their diagnostic performance in a clinical setting.

## MATERIALS AND METHODS

### AFB staining and culture identification

We retrospectively analyzed AFB stain and culture results from clinical specimens performed as standard of care between December 2017 and March 2025 at City of Hope Comprehensive Cancer Center (Duarte, CA, USA). Overall, this retrospective study analyzed 16,294 clinical specimens collected from 7,546 immunocompromised patients. The study protocol was approved by the Institutional Review Board at City of Hope (protocol #25,228).

AFB smears were routinely examined for all specimens submitted for mycobacterial culture, except for blood and intravascular catheter tips. Kinyoun and AR staining were performed on specimen smears according to standard operating procedures and manufacturer’s instructions and following digestion and decontamination for non-sterile sources or directly from normally sterile specimen sources (15). The Kinyoun staining procedure utilized commercially prepared 3% hydrochloric acid/alcohol decolorizer and a stain formula with 2% carbolfuchsin and 5% phenol in reagent alcohol and dimethyl sulfoxide (Medical Chemical Corporation, Torrance, CA, USA). The AR stain kit (Difco Laboratories, Detroit, MI, USA) utilized 0.5% hydrochloric acid/alcohol for decolorization. Kinyoun stained smears were examined under 1,000× oil immersion by light microscopy and AR stain smears under 100× and 400× by fluorescence microscopy. AR stained slides were typically reviewed before Kinyoun stained slides. Both slides were read by the same licensed clinical laboratory scientist, and both stain results were reported. Stain-positive specimens were considered confirmed when both stains were interpreted as AFB-positive, and results were reported semi-quantitatively for each stain (12, 16). Cases of discordance between AR and Kinyoun results were reviewed by a second licensed microbiologist prior to reporting.

Specimens were cultured using Middlebrook 7H10 plates and the Mycobacterial Growth Indicator Tube system (BACTEC MGIT 320, Becton Dickinson). 7H10 plate were incubated at 35°C in 5% CO2 and MGIT vials in the MGIT 320 system until growth or 8 weeks if negative. Growth was confirmed as AFB by Kinyoun stain and subcultured to Lowenstein-Jensen agar and/or 7H10 as needed for isolation and identification. Prior to 2019, AFB isolates were identified by DNA probe for *M. tuberculosis* complex (MTBC) and *M. avium* complex (MAC) (AccuProbe, Hologic Inc., San Diego, CA, USA), otherwise referred for identification by 16s rRNA sequence analysis (ARUP Laboratories, Salt Lake City, UT, USA). Beginning 2019, AFB identification was performed using MTBC and MAC DNA probes or MALDI-TOF mass spectrometry (Vitek MS, bioMérieux, Durham, NC, USA), and starting 2023 was performed solely by MALDI-TOF.

### Diagnostic accuracy analysis

To assess the diagnostic accuracy of Kinyoun and AR staining methods, we calculated sensitivity, specificity, positive predictive value (PPV), negative predictive value (NPV), and likelihood ratios using culture-confirmed results as the reference standard. Briefly, statistical analyses were performed for index cases, defined as the first AFB positive culture specimen from each patient, as well as for all specimens collected. Final results from AFB culture served as the reference standard for smear accuracy. Differences in the performance between Kinyoun and AR stains were evaluated by McNemar’s test, with statistical significance defined at *P* < 0.05. Along with Microsoft Excel, statistical analysis including Poisson and binomial regression analyses for trend evaluation and diagnostic performance with confidence intervals were conducted in R (version 4.5.1).

Semi-quantitative agreement between AR and Kinyoun stains was assessed using weighted Cohen’s *κ* with Fleiss–Cohen to account for ordinal grading differences. Ordinal association was evaluated using Kendall’s *τ*-b. Agreement was summarized as exact match and within ±1 stain grade. Analyses were performed for smear-positive specimens and repeated including not-detected (ND) as the lowest ordinal category. All computations were performed in R (version 4.5.1) using the DescTools and Kendall packages.

## RESULTS

Among 7,546 patients, 54.9% (*n* = 4,146) of them had one specimen tested, while 19.9% (*n* = 1,504) had two, 10.2% (*n* = 768) had three, 5.7% (*n* = 429) had four, 3.3% (*n* = 248) had five specimens tested for AFB culture and stain. An additional 451 patients (6.0%) had six or more stains and cultures performed. Of the total specimens analyzed, 4.1% (668/16,294) yielded positive culture results (Table 1). Among these, 651 cultures recovered *Mycobacterium* spp. and 17 yielded non-mycobacterial organisms such as aerobic Actinomycetes and molds. Among mycobacteria, *Mycobacterium avium* (MAC) complex was the most prevalent (*n* = 366, 56.2%), followed by the *M. tuberculosis* complex (MTBC) (*n* = 94, 14.4%). A total of 557 isolates (85.6%) were identified as NTM, and besides MAC, other frequently detected species included *M. fortuitum* complex (*n* = 40, 6.1%), *M. gordonae* (*n* = 37, 5.7%), *M. abscessus* group (*n* = 27, 4.2%), and *M. chelonae* (*n* = 22, 3.4%). In total, 14.8% (*n* = 96) of the total mycobacterial isolates were classified as RGM (17) (Table 1). Among 336 patients with culture-confirmed mycobacterial cultures, 37 (11.0%) had at least one specimen positive for MTBC, while 88 (26.2%) had two or more positive specimens for the same NTM (Fig. S1). A total of 214 patients (63.7%) had only a single NTM-positive culture, and 3 patients tested positive for both MTBC and NTM (Fig. S1).

**TABLE 1:** Prevalence of *Mycobacterium* spp. and other acid-fast bacilli (AFB) by AFB culture among cancer patients at City of Hope National Medical Center, 2017–2025^*a*^

	All		Index	
**Organisms**	***n***	**%**	***n***	**%**
*Mycobacteria*	651	100	339	100
*Mycobacterium tuberculosis* complex	94	14.44	36	10.62
Nontuberculous mycobacteria (NTM)	557	85.56	303	89.38
Rapid-growing NTM	96	14.75	53	15.63
*Mycobacterium fortuitum* complex	40	6.14	25	7.37
*Mycobacterium abscessus* group	27	4.15	14	4.13
*Mycobacterium chelonae*	22	3.38	9	2.65
*Mycobacterium mucogenicum*	4	0.61	3	0.88
*Mycobacterium neoaurum*	2	0.31	1	0.29
*Mycobacterium smegmatis*	1	0.15	1	0.29
Slow-growing NTM	461	70.81	250	73.75
*Mycobacterium avium* complex	366	56.22	194	57.23
*Mycobacterium gordonae*	37	5.68	27	7.96
*Mycobacterium simiae* complex	24	3.69	12	3.54
*Mycobacterium kansasii*	22	3.38	9	2.65
*Mycobacterium xenopi*	4	0.61	4	1.18
*Mycobacterium arupense, Mycobacterium marinum*	2	0.31	1	0.29
*Mycobacterium lentiflavum, Mycobacterium scrofulaceum*	1	0.15	1	0.29
*Mycobacterium kumamotonense*, *Mycolicibacterium* species	1	0.15		
Non-mycobacterial AFB	17		10	
*Nocardia farcinica*	10		4	
*Nocardia cyriacigeorgica* complex	2		1	
*Gordonia bronchialis*, *Gordonia sputi/aichiensis*, *Nocardia nova* complex, *Nocardia* species, *Nocardia wallacei*	1		1	

^*a*^ All, Includes all specimens analyzed in this study; Index, Refers to the first culture positive specimen collected from each patient.

Among culture-positive mycobacteria (*n =* 651), the majority were obtained from respiratory samples (44.2% sputum and 43.3% other respiratory) (Table S1). Among index mycobacterial specimens, non-sputum respiratory samples (predominantly bronchoalveolar lavage fluids) were the most common source (59.6%), followed by sputum (23.9%). Other specimen types such as skin, soft tissue, body fluids, stool, cerebrospinal fluid, urine, and bone marrow contributed to smaller proportions (Table S1).

**Fig 1:**
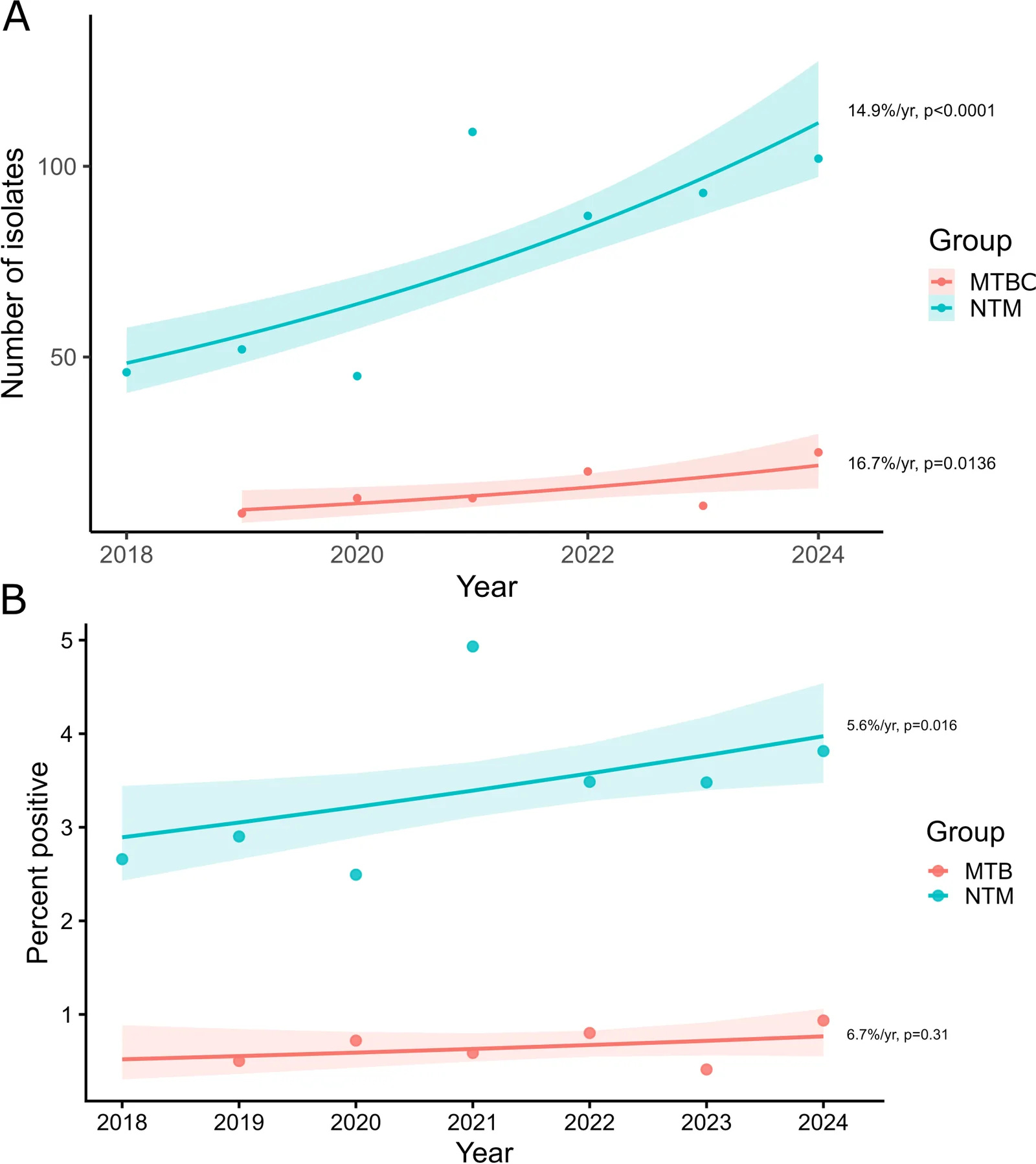
Trends in isolation number and percent positivity and isolation rates for *M. tuberculosis* complex (MTBC) and nontuberculous mycobacteria (NTM) (2018–2024). (**A**) Number of mycobacteria isolated each year. Isolation trend modeled using a Poisson fit with 95% CI. MTBC (red) shows an increase of 16.7%/year, while NTM (blue) shows an increase of 14.9%/year. Shaded areas represent 95% CI. (**B**) Percent of culture positive mycobacteria among all specimens tested. Percent positivity trend modeled using a binomial fit with 95% confidence intervals (CI). The red line represents MTBC (6.7%/year), and the blue line represents NTM (5.7%/year). Shaded areas indicate 95% CI.

Between 2018 and 2024 (data encompassing complete years during the study period), a significant upward trend was observed in the number of cultures recovering MTBC (14.9% increase, *P* < 0.0001) and NTM (16.7% increase, *P* = 0.01) within our patient population (Fig. 1A). The rate of positive isolation also demonstrated an increasing trend over time for both MTBC (6.7%, *P* = 0.31) and NTM (5.6%, *P* = 0.02) (Fig. 1B). Additionally, we observed a marked increase in both the number and frequency of NTM isolates during 2021.

### Diagnostic accuracy of AR and Kinyoun stain in detecting mycobacteria

Across all specimens and index cases, AR and Kinyoun stains both demonstrated high specificity (>99.9%) (Tables 2 and 3) with only one case in which a positive stain corresponded to no culture growth. Among 17 culture positive for aerobic Actinomycetes, including *Gordonia* and *Nocardia* spp., one *Nocardia* spp*.* was detected by Kinyoun stain. AR stain trended toward higher sensitivity for MTBC strains (43.6%, CI: 34.0–53.7) compared to Kinyoun (40.4%, CI: 31.1–50.5) and Kinyoun marginally outperformed AR for NTM strains (13.5%, CI: 10.9–16.6 vs 12.9%, CI: 10.4–16.0) (Table 2), but there was substantial overlap in 95% confidence intervals. Among index cases, MTBC detection was equivalent for both stains (16.7%, CI: 7.9–31.9), and NTM detection was similar between Kinyoun (11.2%, CI: 8.1–15.3) and AR (10.6%, CI: 7.6–14.5) (Table 3).

**TABLE 2:** Diagnostic accuracy of auramine-rhodamine and Kinyoun staining across all specimens, with 95% confidence intervals^*a*^

Metrics	MTBC-AR	MTBC-K	NTM-AR	NTM-K	RGM-AR	RGM-K	SGNTM-AR	SGNTM-K
Sensitivity	43.62	40.43	12.93	13.46	16.67	21.88	12.15	11.71
95% CI	34.04–53.70	31.07–50.53	10.39–15.97	10.88–16.55	10.53–25.37	14.78–31.14	9.47–15.45	9.09–14.97
Specificity	99.99	99.99	99.99	99.99	99.99	99.99	99.99	99.99
95% CI	99.96–100.00	99.96–100.00	99.96–100.00	99.96–100.00	99.96–100.00	99.96–100.00	99.96–100.00	99.96–100.00
PPV	97.62	97.44	98.63	98.68	94.12	95.45	98.25	98.18
95% CI	87.68–99.58	86.82–99.55	92.64–99.76	92.92–99.77	73.02–98.95	78.20–99.19	90.71–99.69	90.39–99.68
NPV	99.66	99.64	96.99	97.00	99.49	99.52	97.47	97.46
95% CI	99.56–99.74	99.54–99.72	96.71–97.24	96.73–97.26	99.37–99.59	99.40–99.62	97.22–97.70	97.20–97.69
LR+	6,809.49	6,311.23	2,018.07	2,102.15	2,602.00	3,415.12	1,896.47	1,828.74
95% CI	946.47– 48,991.63	875.58– 45,492.02	280.95– 14,495.79	292.82– 15,091.61	348.54– 19,425.29	464.03– 25,134.50	263.10– 13,670.08	253.54– 13,190.34
LR-	0.56	0.60	0.87	0.87	0.83	0.78	0.88	0.88
95% CI	0.47–0.67	0.50–0.70	0.84–0.90	0.84–0.89	0.76–0.91	0.70–0.87	0.85–0.91	0.85–0.91

^*a*^ MTBC, *M. tuberculosis* complex; NTM, nontuberculous mycobacteria; RGM, rapid-growing mycobacteria; SGNTM, slow-growing NTM; AR, auramine-rhodamine; K, Kinyoun staining; PPV, positive predictive value; NPV, negative predictive value; LR+, positive likelihood ratios; and LR−, negative likelihood ratios, each with corresponding 95% confidence intervals (CI).

**TABLE 3:** Diagnostic accuracy of auramine-rhodamine and Kinyoun staining across index specimens, with 95% confidence intervals^*a*^

Metrics	MTBC-AR	MTBC-K	NTM-AR	NTM-K	RGM-AR	RGM-K	SGNTM-AR	SGNTM-K
Sensitivity	16.67	16.67	10.56	11.22	11.32	18.87	10.40	9.60
95% CI	7.87–31.89	7.87–31.89	7.58–14.53	8.14–15.27	5.29–22.58	10.59–31.36	7.20–14.80	6.54–13.89
Specificity	99.99	99.99	99.99	99.99	99.99	99.99	99.99	99.99
95% CI	99.92–100.00	99.92–100.00	99.92–100.00	99.92–100.00	99.92–100.00	99.92–100.00	99.92–100.00	99.92–100.00
PPV	85.71	85.71	96.97	97.14	85.71	90.91	96.30	96.00
95% CI	48.69–97.43	48.69–97.43	84.68–99.46	85.47–99.49	48.69–97.43	62.26–98.38	81.72–99.34	80.46–99.29
NPV	99.60	99.60	96.48	96.51	99.37	99.43	97.08	97.05
95% CI	99.43–99.72	99.43–99.72	96.05–96.87	96.08–96.90	99.17–99.53	99.23–99.57	96.67–97.43	96.65–97.41
LR+	1,239.50	1,239.50	785.43	834.51	841.92	1,403.21	773.45	713.95
95% CI	153.08– 10,036.55	153.08– 10,036.55	107.68– 5,728.80	114.62– 6,076.04	103.13– 6,873.18	182.86– 10,767.75	105.38– 5,677.00	96.97– 5,256.51
LR−	0.83	0.83	0.89	0.89	0.89	0.81	0.90	0.90
95% CI	0.72–0.96	0.72–0.96	0.86–0.93	0.85–0.92	0.81–0.98	0.71–0.92	0.86–0.93	0.87–0.94

^*a*^ MTBC, *M. tuberculosis* complex; NTM, nontuberculous mycobacteria; RGM, rapid-growing mycobacteria; SGNTM, slow-growing NTM; AR, auramine-rhodamine; K, Kinyoun staining; PPV, positive predictive value; NPV, negative predictive value; LR+, positive likelihood ratios; and LR−, negative likelihood ratios, each with corresponding 95% confidence intervals (CI).

By specimen source, overall detection of MTBC was higher with AR than Kinyoun in both sputum (65.8% vs 60.5%) (*P* = 0.48) and other respiratory sample types (41.7% vs 38.9%) (*P =* 1.0) (Table S2), though differences were not statistically significant. For NTM, detection rates were low overall, with minimal difference between AR and Kinyoun stains in sputum (10.0% vs 11.2%, respectively) (*P* = 0.25) and other respiratory samples (16.3% vs 15.4%, respectively) (*P* = 0.62). While neither stain detected MTBC in skin and soft tissue samples, both were able to detect a small proportion of NTM in these sources: 17.1% (AR) and 20.0% (Kinyoun) (*P* = 1.0).

By organism group, there were four index cases in which RGM isolates (*M. abscessus* group, *M. chelonae*, *M. fortuitum* complex) were identified only by Kinyoun stain, while there were two index cases in which slow-growing mycobacteria (MAC) were detected exclusively by AR (Table 4; Table S3a). Of the four AR−/Kinyoun+ RGM cases, two (one *M. abscessus,* one *M. chelonae*) originated from sites of skin and soft tissue infection, deemed clinically significant, and treated, while two (one *M. fortuitum*, one *M. chelonae*) were from bronchoalveolar lavage fluids in patients with alternative infectious diagnoses, interpreted as clinically insignificant by infectious diseases consultants, and not treated. The two AR+/Kinyoun− MAC cases were considered pulmonary MAC disease based on clinical and radiological findings and treated by infectious diseases physicians. Across all specimens, Kinyoun uniquely detected five (5.2%) RGM isolates out of 96 culture-positive RGM cases, including *M. abscessus* group, *M. chelonae*, *M. fortuitum* complex, and *M. neoaurum*, whereas AR uniquely detected 6 (1.1%) slow-growing mycobacteria (SGM) isolates out of 555 SGM-positive cases, including MAC and MTBC. AR staining identified three additional MTBC isolates in follow-up smears compared to Kinyoun stain (*P* = 0.25). Although the trend is statistically not significant, Kinyoun staining showed higher sensitivity than AR for RGM across all specimens (21.9% vs 16.7%, *P* = 0.07) and index cases (18.9% vs 11.3%, *P* = 0.13) (Tables 2 and 3). Slow-growing NTM detection was similar between AR and Kinyoun across all specimens (12.2% vs 11.7%, *P* = 0.48) and index cases (10.4% vs 9.6%, *P* = 0.48) (Tables 2 and 3).

**TABLE 4:** Detection of *Mycobacterium* spp. in index and all specimens using auramine-rhodamine (AR) and/or Kinyoun staining methods

	AR+ and Kinyoun−		AR− and Kinyoun+		Total
	Organisms	*n*	Organisms	*n*	
Index specimen	*M. avium* complex	2	*M. abscessus* group	1	6
			*M. chelonae*	2	
			*M. fortuitum* complex	1	
		(2)		(4)	
All specimen	*M. avium* complex	3	*M. abscessus* group	1	12
	*M. tuberculosis* complex	3	*M. avium* complex	1	
			*M. chelonae*	2	
			*M. fortuitum* complex	1	
			*M. neoaurum*	1	
		(6)		(6)	

Overall, both stains demonstrate excellent predictive values, with PPV slightly higher for NTM (>98.6%) than MTBC (>97.4%), while NPV was slightly higher for MTBC (>99.6%) compared to NTM (>97.0%). Positive likelihood ratios were higher for MTBC (AR: 6,810; Kinyoun: 6,311) than NTM (AR: 2,018; Kinyoun: 2,102), and negative likelihood ratios remained low (MTBC: 0.6 for AR, 0.6 for Kinyoun; NTM: 0.9 for both) (Table 2). Among index cases, NTM showed slightly better PPV (>97.0%) than MTBC (85.7%) for both stains. NPV was consistently high for MTBC (99.6%) and NTM (97.5%). Likelihood ratios followed a similar trend, with LR+ highest for MTBC (1,240) and lower for NTM (AR: 785; Kinyoun: 835), while LR− remained low (0.8 for MTBC; 0.9 for NTM) (Table 3). Overall, nine species (56.3%) were among index cases, and nine species (53.0%) across all specimens were not detected by either staining method (Table S3a and b).

### Comparative semi-quantification of AR and Kinyoun stains toward mycobacterial groups

**Fig 2:**
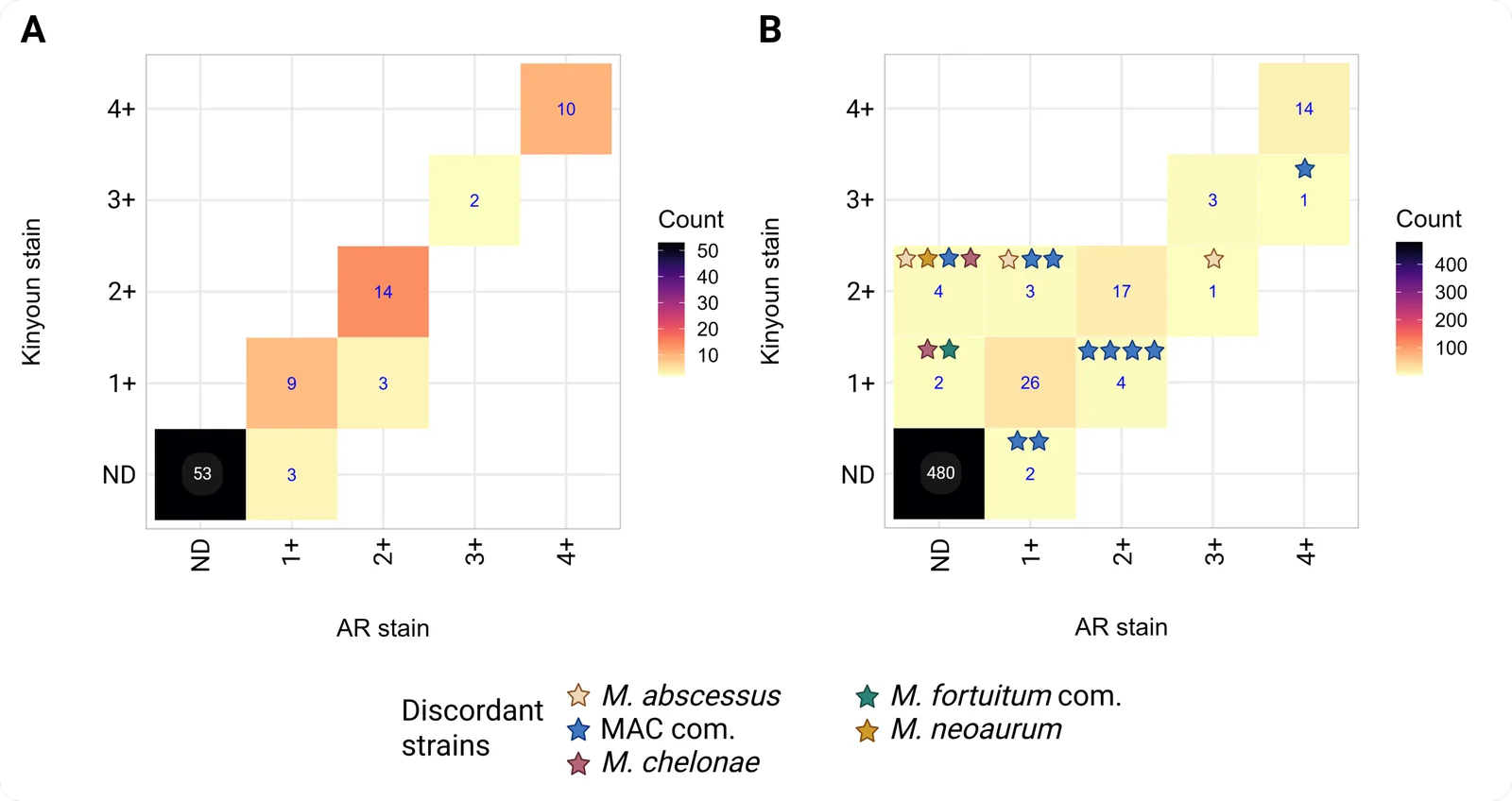
Comparative diagnostic performance of auramine-rhodamine and Kinyoun staining for grading *M. tuberculosis* complex (MTBC, **A**) and nontuberculous mycobacteria (NTM, **B**). MAI; *Mycobacterium avium* complex.

Semi-quantitative results of AR and Kinyoun stains among 107 AR+/Kinyoun+ smears were evaluated for concordance (Fig. 2A). Among smear-positive MTB specimens, weighted Cohen’s *κ* (Fleiss-Cohen) was 0.97, indicating strong agreement between AR and Kinyoun semi-quantitative grades. Kendall’s *τ*-b was 0.93 (*P* < 0.001), confirming strong ordinal association. Exact agreement occurred in 92.1% of pairs, and all pairs (100%) were within ±1 grade. When “not detected” (ND) results were included as the lowest ordinal category, agreement further increased (weighted *κ* = 0.98; Kendall’s *τ*-b = 0.95 (*P* < 0.001)), with 93.6% exact agreement and no multi-step differences. Among smear-positive NTM specimens, weighted Cohen’s *κ* was 0.95, indicating strong agreement between AR and Kinyoun semi-quantitative grades. Kendall’s *τ*-b was 0.88 (*P* < 0.001), confirming strong ordinal association. Exact agreement occurred in 87.0% of pairs, and all pairs were within ±1 grade. When ND was incorporated as the lowest ordinal category, agreement further increased: weighted *κ* was 0.96. Kendall’s *τ*-b was 0.93 (*P* < 0.001). Exact agreement rose to 97.0% and 99.3% of pairs were within ±1 grade. Specifically, among dual AR and Kinyoun smear-positive NTM, AR stained specimens one grade higher than Kinyoun in six cases (5 MAC, 1 RGM), whereas Kinyoun stained specimens one grade higher than AR in three cases (2 MAC, 1 RGM) (Fig. 2B). Among AR and Kinyoun discordant results, AR scored smears 1+ in two cases that were Kinyoun-negative, while Kinyoun labeled smears as 1+ in two cases and 2+ in four cases with AFB that were undetected by AR. For MTBC, the most common semi-quantification categories were few (2+) and many (4+), but for NTM were rare (1+) and few (2+) (Fig. 2).

## DISCUSSION

Tuberculosis caused by *Mycobacterium tuberculosis* remains one of the leading communicable diseases globally, accounting for 1.25 million deaths and 10.8 million active cases worldwide in 2023 (18). According to CDC, 9,633 cases of TB infection were reported in the USA in 2023, while ~13 million people harbor latent TB infection (19). Immunodeficient patients with hematological malignancy have 2–40 times higher risk of progressing to TB disease once infected and experience higher mortality rates (20). Apart from MTB, MAC and *Mycobacterium kansasii,* as well as RGM, particularly *Mycobacterium abscessus* complex, may cause serious pulmonary infection in immunocompromised patients (21). Diagnostic testing for AFB remains critical in immunocompromised patients who are susceptible to a broad range of pathogens that may present similarly.

AFB smear microscopy remains the primary method to rapidly detect mycobacteria from patient specimens (12). Guidelines including from the National Center for Infectious Diseases, CDC (22) and American Thoracic Society/Infectious Diseases Society of America (23, 24), and others (25) recommend fluorochrome-based staining (AR, AO) over carbolfuchsin stain (Kinyoun, ZN) for AFB smear staining. However, few studies have directly compared these stains for NTM detection in primary specimen screening, particularly in immunocompromised patients.

In this retrospective review of 16,294 specimens over 7 years, both Kinyoun and AR stains demonstrated high specificity (>99%) but relatively low sensitivity (17% overall, 11% among index cases). The difference in sensitivity observed for index cases could be attributable to the exclusion follow-up specimens, which are often performed on smear-positive cases until conversion to smear-negative in alignment with clinical guidelines (26, 27). Our study also confirmed the importance of NTM in our patient population which accounted for 85% of AFB isolates, most of which were MAC or RGM. Neither stain detected any of the 27 *M. gordonae* and 12 *M. simiae* complex cases, likely due to low organism loads particularly as *M. gordonae* is a well-established contaminant and *M. simiae* is often considered clinically non-significant (24, 28). Other studies have also substantiated the lower detection rate of AFB smears for *M. gordonae* though data on smear positivity with *M. simiae* are sparse outside of clinically significant cases (29). Furthermore, extrapulmonary specimens, such as CSF from patients with tuberculosis meningitis (30) or pleural effusion (31), typically contain fewer bacilli than respiratory samples (32). AFB smear microscopy also requires a relatively high organism burden for detection (~5,000–10,000 bacilli/mL) (33, 34). This lower bacillary load likely contributed to the overall reduced sensitivity observed in extrapulmonary specimens by both AR and Kinyoun stains in our data set.

Performance differences between AR and Kinyoun stains were small and statistically non-significant. AR staining demonstrated a trend towards higher sensitivity for MTBC overall (43.6% vs 40.4%), but similar sensitivity among index cases (16.7%). Kinyoun staining showed comparable sensitivity to AR for NTM (13.5% vs 12.9%) but trended toward higher sensitivity for RGM (21.9% vs 16.7%). Semi-quantitative grading between AR and Kinyoun methods had high concordance though AR tended toward higher grading of MAC and MTB and Kinyoun tended toward higher grading for RGM in a small number of cases. The comparable results between AR and Kinyoun stains also support the use of the cold-staining Kinyoun as a safe, practical alternative for AFB smear microscopy in resource-limited settings lacking access to fluorescence microscopy or adequate biosafety facilities.

These observations align with prior reports indicating enhanced performance of auramine-based stains for MTBC and contribute to the paucity of data on the relative performance of fluorochrome and carbolfuschin stains for NTM. In other head-to-head comparisons, fluorochrome consistently outperformed carbolfuschin-based stains in the detection of MTBC from clinical specimens (35–37), with a meta-analysis finding that sensitivity was about 10% higher (11). Another study found AO to be superior to ZN in histologic sections for detection of NTM (14). However, other studies have found that cultured RGM isolates do not consistently stain positively with AO or AR stains as they do with ZN stain, raising concerns of relying solely on AR stain for AFB smear testing (13, 38). Furthermore, not all cells of some isolates stained acid-fast, thereby decreasing the theoretical limit of detection by AFB staining in such RGM. These findings were described in isolates that had been passaged in the laboratory (13), whereas our study provides direct stain comparisons using clinical specimens. While we identified RGM cases that had Kinyoun-positive/AR-negative smears or higher grading by Kinyoun than AR stain, the differences were small and seen in a small number of cases over the 7-year study period. Notably, Kinyoun stain detected only two clinically significant RGM cases missed by AR and both from skin and soft tissue infection sites.

Others have also found AFB smears to have higher detection rates for MTBC than for NTM (23, 29). The Kinyoun staining method has reported sensitivities of 53%–82% for the detection of MTBC when compared to culture (39, 40). The performance of AO or AR staining to detect MTBC has been similarly variable between published studies, with a detection rate of 43% in sputum compared to culture, according to 2010 CDC data (41). Others have found higher sensitivities in the range of 56.3%–93.1% (42, 43).

Our study unexpectedly identified local epidemiological trends including a surge in NTM cases in 2021. Temporally, it could be related to the COVID-19 pandemic and its associated effects on the community’s access of healthcare, interaction with environmental sources of pathogens, and other factors (44). Regardless, little epidemiological data have been published to correlate our findings though a steady global increase in NTM infections has been described over recent years (45). MTB accounted for 14.5% of AFB isolated at our institution, consistent with previous studies which reported a TB prevalence of approximately 12.3% of AFB infections among cancer patients (46). In contrast, earlier reports indicated that 29.7%–47% of isolated NTM cases in cancer patients were due to MAC while our study identified a higher proportion of MAC (65.7%) (47–49).

Our data should be interpreted in light of the patient population tested, as the yield of AFB smear and culture is highly dependent on host immune status, mycobacterial species, and laboratory technique, all of which can affect bacillary load in the specimen to be tested. The majority of patients seen at our facility are non-HIV, immunocompromised individuals who present for cancer care rather than primary diagnosis of mycobacterial disease. Specimens were mainly from the lower respiratory tract and bronchoalveolar lavages are frequently performed for infectious work-up with AFB smear and culture routinely included in the battery of testing. Our laboratory has used Kinyoun instead of ZN as the primary carbolfuschin AFB stain. Others have described inferior performance of Kinyoun compared to ZN staining (25), though staining performance is subject to numerous technical variables (50, 51), and we showed that in our hands, Kinyoun compared nearly equivalently to AR. As a retrospective study, however, we were unable to control for potential bias in smear reading, as a positive AFB stain by one method may influence the reading of the other. We focused on AFB stain performance compared to culture but note that not all NTM isolates are considered significant, acknowledging that clinical and radiological correlations are required to confirm mycobacterial disease.

In summary, our findings underscore some subtleties in mycobacterial organism-specific stain performance, but overall high concordance between AR and Kinyoun stains of specimen smears. AR staining performed somewhat better for MTBC and slow-growing species, and Kinyoun staining tended toward a higher sensitivity trend for RGM, but neither finding was statistically significant. Although we show that it is possible to miss a small number of RGM cases if only using AR for primary AFB staining, smears should be complemented by culture to maximize detection of AFB. In a population with predominantly NTM isolation from AFB culture, AR staining was adequate as a primary method to detect mycobacteria, but Kinyoun staining could be considered an additional stain when RGM is suspected or in resource-limited settings.

## SUPPLEMENTAL MATERIAL

Data S1 spectrum.00475-26-s0001.xlsx De-identified laboratory data set of acid-fast bacilli cultures spanning multiple years, including specimen source and type, patient age, Kinyoun and AR stain results with grading, and corresponding culture identifications.

Supplemental material spectrum.00475-26-s0002.docx Tables S1 to S3; Fig. S1.

ASM does not own the copyrights to Supplemental Material that may be linked to, or accessed through, an article. The authors have granted ASM a non-exclusive, world-wide license to publish the Supplemental Material files. Please contact the corresponding author directly for reuse.
