# Supplementary material for: Comparison of diagnostic accuracy and sensitivity of Kinyoun and auramine-rhodamine stains in detecting mycobacteria from specimen smears
Source: Microbiol Spectr. 2026 May 19;14(7):e00475-26. doi: 10.1128/spectrum.00475-26 (PMC13339896; doi:10.1128/spectrum.00475-26)
Supplement: Supplemental material — Tables S1 to S3; Fig. S1. [file spectrum.00475-26-s0002.docx]

**Table S1:** Distribution of culture-positive mycobacterial isolates by specimen types among cancer patients.

|  | **All** | | **Index** | |
| --- | --- | --- | --- | --- |
| **Specimen type** | **n** | **%** | **n** | **%** |
| Sputum | 288 | 44.24 | 81 | 23.89 |
| Non sputum respiratory | 282 | 43.32 | 202 | 59.59 |
| Skin & soft tissue | 48 | 7.37 | 32 | 9.44 |
| Body fluid | 12 | 1.84 | 9 | 2.65 |
| Stool | 12 | 1.84 | 9 | 2.65 |
| Urine | 4 | 0.61 | 2 | 0.59 |
| Cerebrospinal fluid | 3 | 0.46 | 3 | 0.88 |
| Bone marrow | 1 | 0.15 |  |  |
| Other | 1 | 0.15 | 1 | 0.29 |

All; Includes all specimens analyzed in this study, Index; Refers to the first culture positive specimen collected from each patient.

**Table S2:** Percentage detection of culture positive mycobacteria by Kinyon and auramine-rhodamine staining across specimen types.

|  |  |  | **% of positive detection (+/total)** | | | |
| --- | --- | --- | --- | --- | --- | --- |
|  |  | | **MTBC** | | **NTM** | |
| **Specimen types** | **All**  **n** | **Culture +**  **n, (%)** | **Kinyoun** | **AR** | **Kinyoun** | **AR** |
| Body Fluid | 2309 | 12 (0.52) | 33.33 (1/3) | 33.33 (1/3) | 11.11 (1/9) | 0 (0/9) |
| Bone Marrow | 29 | 1 (3.5) |  |  | 0 (0/1) | 0 (0/1) |
| Cerebrospinal Fluid | 1624 | 3 (0.18) | 0 (0/1) | 0 (0/1) | 0 (0/2) | 0 (0/2) |
| Non sputum respiratory | 7557 | 282 (3.7) | 38.88 (14/36) | 41.66 (15/36) | 15.44 (38/246) | 16.26 (40/246) |
| Skin & soft tissue | 2036 | 48 (2.4) | 0 (0/13) | 0 (0/13) | 20.00 (7/35) | 17.14 (6/35) |
| Sputum | 1712 | 288 (16.8) | 60.52 (23/38) | 65.78 (25/38) | 11.20 (28/250) | 10.00 (25/250) |
| Stool | 72 | 12 (16.7) | 0 (0/1) | 0 (0/1) | 0 (0/11) | 0 (0/11) |
| Urine | 134 | 4 (3.0) | 0 (0/2) | 0 (0/2) | 0 (0/2) | 0 (0/2) |
| Other | 821 | 1 (0.1) |  |  | 100 (1/1) | 100 (1/1) |

MTBC; *M. tuberculosis* complex, NTM; nontuberculous mycobacteria, AR; auramine-rhodamine staining

**Table S3a**: Detection of *Mycobacterium* spp. in index specimens using auramine-rhodamine (AR) and/or Kinyoun staining methods.

| **Stain** | **AR+** | | **AR-** | | **Total** |
| --- | --- | --- | --- | --- | --- |
|  | **Organisms** | **n** | **Organisms** | **n** |  |
| **Kinyoun+** | *M. abscessus* group | 4 |  |  | 40 |
|  | *M. avium* complex | 22 |  |  |  |
|  | *M. chelonae* | 1 | *M. abscessus* group | 1 |  |
|  | *M. fortuitum* complex | 1 | *M. chelonae* | 2 |  |
|  | *M. kansasii* | 1 | *M. fortuitum* complex | 1 |  |
|  | *M. marinum* | 1 |  |  |  |
|  | *M. tuberculosis* complex | 6 |  |  |  |
|  |  | (36) |  | (4) |  |
| **Kinyoun-** |  |  | *M. abscessus* group | 9 | 299 |
|  |  |  | *M. avium* complex | 170 |  |
|  |  |  | *M. chelonae* | 6 |  |
|  |  |  | *M. fortuitum* complex | 23 |  |
|  |  |  | *M. kansasii* | 8 |  |
|  |  |  | *M. neoaurum* | 1 |  |
|  |  |  | *M. simiae* complex | 12 |  |
|  |  |  | *M. tuberculosis* complex | 30 |  |
|  |  |  | *M. arupense* | 1 |  |
|  | *M. avium* complex | 2 | *M. gordonae* | 27 |  |
|  |  |  | *M. lentiflavum* | 1 |  |
|  |  |  | *M. mucogenicum* | 3 |  |
|  |  |  | *M. scrofulaceum* | 1 |  |
|  |  |  | *M. smegmatis* | 1 |  |
|  |  |  | *M. xenopi* | 4 |  |
|  |  | (2) |  | (297) |  |
| **Total** |  | 38 |  | 301 | 339 |

**Table S3b:** Detection of *Mycobacterium* spp. in all specimens using auramine-rhodamine (AR) and/or Kinyoun staining methods.

| **Stain** | **AR+** | | **AR-** | | **Total** |
| --- | --- | --- | --- | --- | --- |
|  | **Organisms** | **n** | **Organisms** | **n** |  |
| **Kinyoun+** | *M. abscessus* group | 11 |  |  | 113 |
|  | *M. avium* complex | 47 |  |  |  |
|  | *M. chelonae* | 4 | *M. abscessus Group* | 1 |  |
|  | *M. fortuitum* complex | 1 | *M. avium* complex | 1 |  |
|  | *M. kansasii* | 4 | *M. chelonae* | 2 |  |
|  | *M. marinum* | 1 | *M. fortuitum* complex | 1 |  |
|  | *M. simiae* complex | 1 | *M. neoaurum* | 1 |  |
|  | *M. tuberculosis* complex | 38 |  |  |  |
|  |  | (107) |  | (6) |  |
| **Kinyoun-** |  |  | *M. abscessus* group | 15 | 538 |
|  |  |  | *M. avium* complex | 315 |  |
|  |  |  | *M. chelonae* | 16 |  |
|  |  |  | *M. fortuitum* complex | 38 |  |
|  |  |  | *M. kansasii* | 18 |  |
|  |  |  | *M. marinum* | 1 |  |
|  |  |  | *M. neoaurum* | 1 |  |
|  |  |  | *M. simiae* complex | 23 |  |
|  |  |  | *M. tuberculosis* complex | 53 |  |
|  | *M. avium* complex | 3 | *M. arupense* | 2 |  |
|  | *M. tuberculosis* complex | 3 | *M. gordonae* | 37 |  |
|  |  |  | *M. kumamotonense* | 1 |  |
|  |  |  | *M. lentiflavum* | 1 |  |
|  |  |  | *M. mucogenicum* | 4 |  |
|  |  |  | *M. scrofulaceum* | 1 |  |
|  |  |  | *M. smegmatis* | 1 |  |
|  |  |  | *M. xenopi* | 4 |  |
|  |  |  | *M.* species | 1 |  |
|  |  | (6) |  | (532) |  |
| **Total** |  | 113 |  | 538 | 651 |


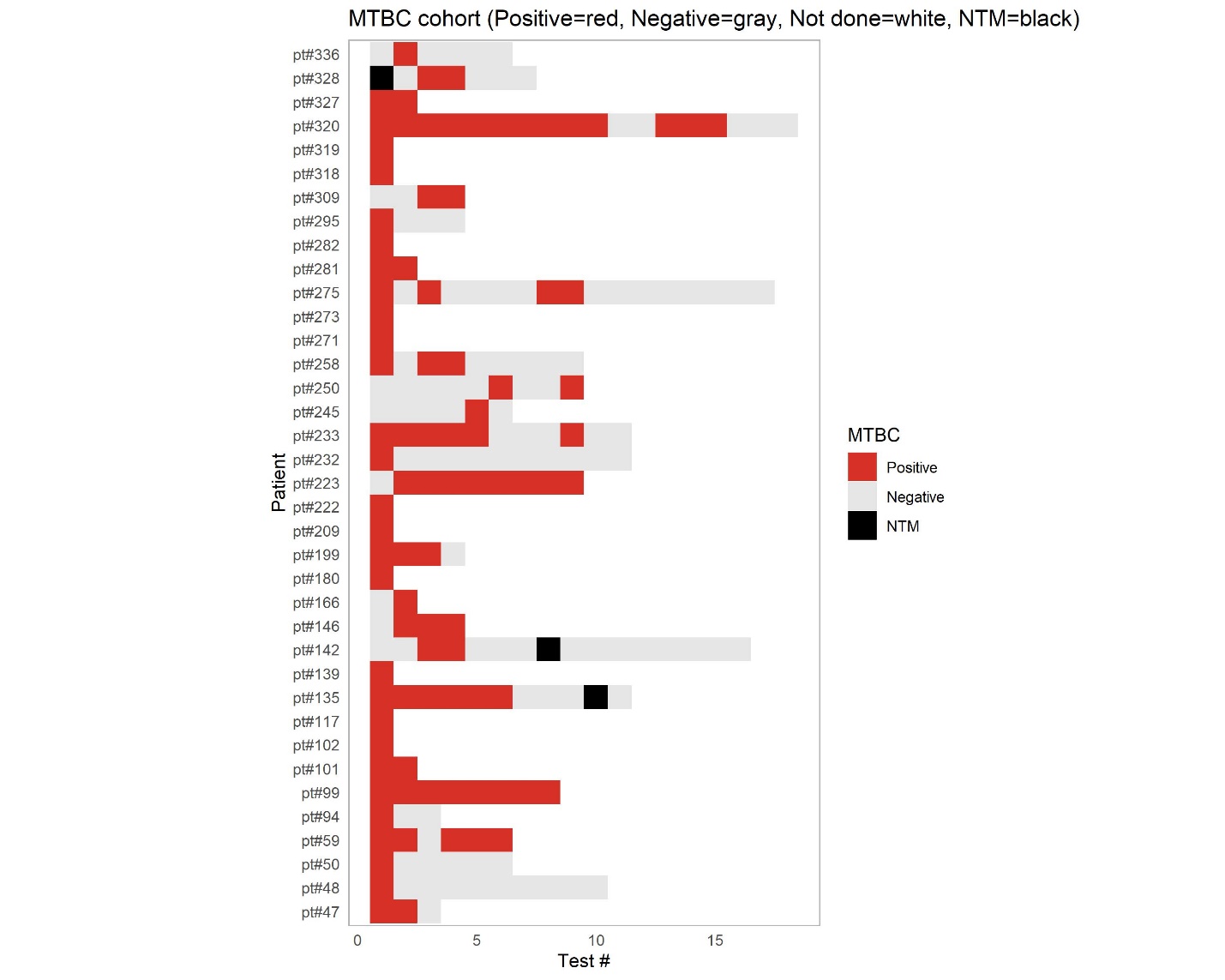


A

B


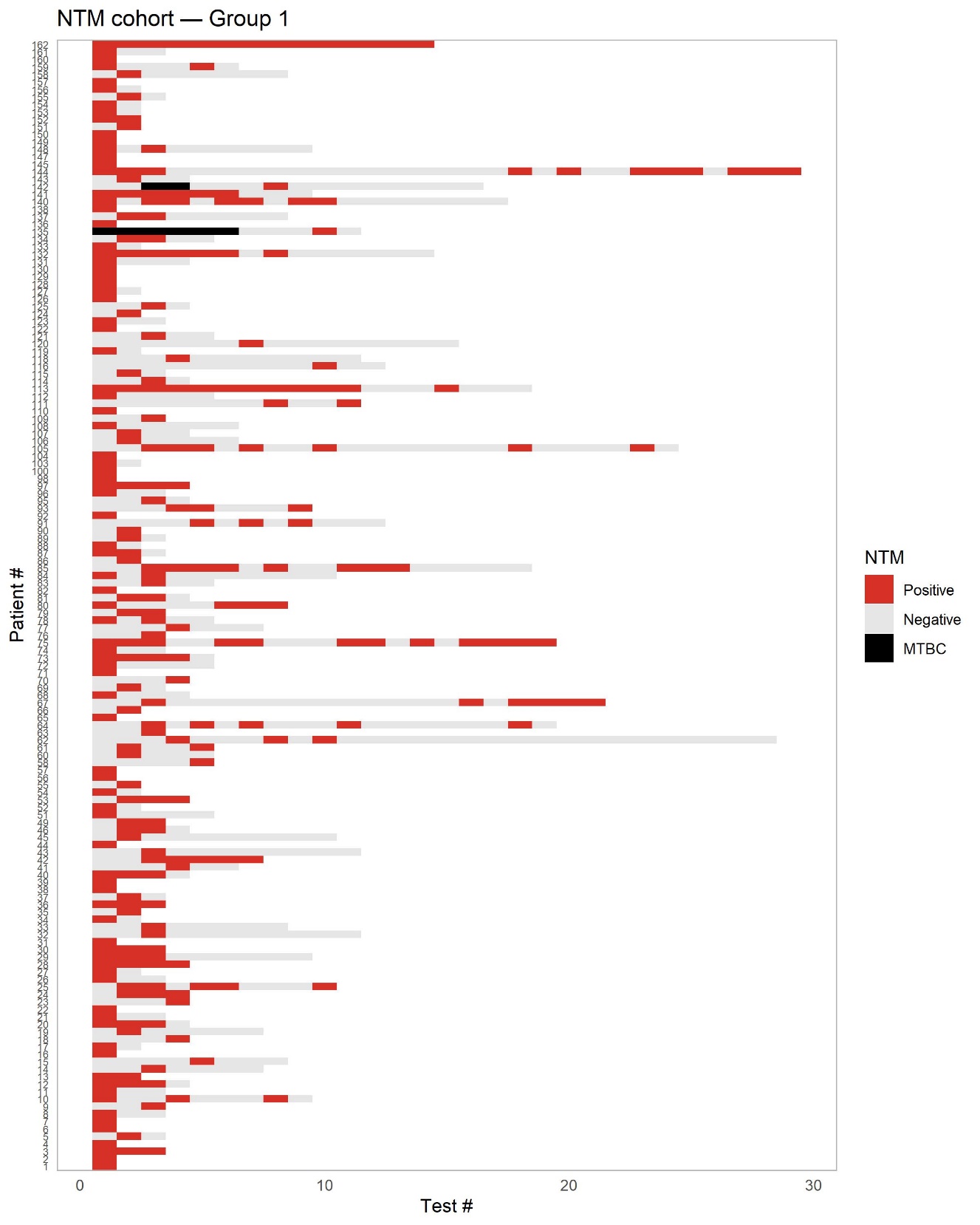


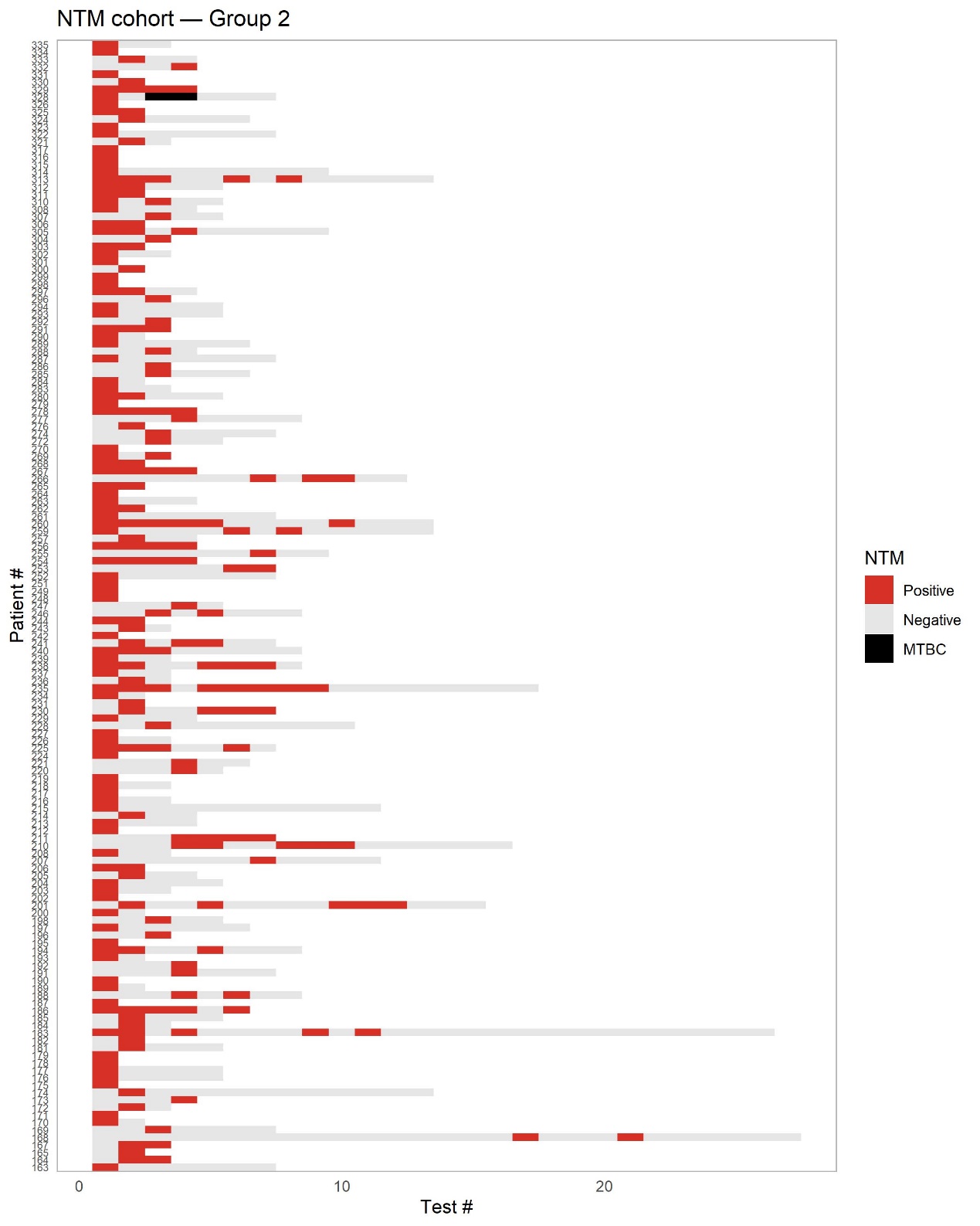


C

**Figure S1.** Detection of *Mycobacterium* spp. across culture-positive patients. Panels A–C show the distribution of culture-positive results for *M. tuberculosis* complex (MTBC, A) and nontuberculous mycobacteria (NTM, B–C). Each row represents an individual patient, and each column corresponds to a test instance. Color coding indicates test outcomes: red = positive, gray = negative, and white = test not performed or other mycobacteria. The x-axis shows sequential test numbers, and the y-axis lists patient IDs. This visualization highlights the frequency and pattern of MTBC and NTM detection across the cohort.
